# The Presence of Toxic and Non-Toxic Cyanobacteria in the Sediments of the Limpopo River Basin: Implications for Human Health

**DOI:** 10.3390/toxins10070269

**Published:** 2018-07-03

**Authors:** Murendeni Magonono, Paul Johan Oberholster, Addmore Shonhai, Stanley Makumire, Jabulani Ray Gumbo

**Affiliations:** 1 Department of Hydrology and Water Resources, School of Environmental Sciences, University of Venda, Thohoyandou 0950, South Africa; murendy22@gmail.com; 2 Council for Scientific and Industrial Research, Natural Resources and the Environment, Stellenbosch 7600, South Africa; poberholster@csir.co.za; 3 Department of Biochemistry, School of Mathematical and Natural Sciences, University of Venda, Thohoyandou 0950, South Africa; addmore.shonhai@univen.ac.za (A.S.); stanmakster@gmail.com (S.M.)

**Keywords:** cyanobacteria, cyanotoxins, nutrient enrichment, akinetes, harmful algal blooms, PCR, phylogenetic analyses

## Abstract

The presence of harmful algal blooms (HABs) and cyanotoxins in drinking water sources poses a great threat to human health. The current study employed molecular techniques to determine the occurrence of non-toxic and toxic cyanobacteria species in the Limpopo River basin based on the phylogenetic analysis of the 16S rRNA gene. Bottom sediment samples were collected from selected rivers: Limpopo, Crocodile, Mokolo, Mogalakwena, Nzhelele, Lephalale, Sand Rivers (South Africa); Notwane (Botswana); and Shashe River and Mzingwane River (Zimbabwe). A physical-chemical analysis of the bottom sediments showed the availability of nutrients, nitrates and phosphates, in excess of 0.5 mg/L, in most of the river sediments, while alkalinity, pH and salinity were in excess of 500 mg/L. The FlowCam showed the dominant cyanobacteria species that were identified from the sediment samples, and these were the *Microcystis* species, followed by *Raphidiopsis raciborskii*, *Phormidium* and *Planktothrix* species. The latter species were also confirmed by molecular techniques. Nevertheless, two samples showed an amplification of the cylindrospermopsin polyketide synthetase gene (S3 and S9), while the other two samples showed an amplification for the microcystin/nodularin synthetase genes (S8 and S13). Thus, these findings may imply the presence of toxic cyanobacteria species in the studied river sediments. The presence of cyanobacteria may be hazardous to humans because rural communities and farmers abstract water from the Limpopo river catchment for human consumption, livestock and wildlife watering and irrigation.

## 1. Introduction

Toxic and non-toxic cyanobacteria species are on the increase in most parts of the world, including in South Africa. The emergence and resurgence of harmful algal blooms (HABS) is due to eutrophication. The toxic cyanobacteria are known to carry genes that produce cyanotoxins which are lethal to humans. However, the toxic and non-toxic cyanobacteria species merely differ in the *mcy* gene content, which is the peptide synthetase producing microcystin [1]. This may explain the observation of non-detectable microsystin toxin despite the presence of *mcy* gene [2]. A study by Frazao et al. [3] used the PCR method to determine molecular analysis of genes involved in the production of known cyanotoxins, microcystins, nodularins and cylindrospermopsin. The toxic strains of the cyanobacteria genera, *Leptolyngbya*, *Oscillatoria*, *Microcystis*, *Planktothrix* and *Anabaena* are known to have in common the *mcy* (*A–E*, *G*, *J*) genes that are involved in the biosynthesis of microcystin [1,3]. The nodularin cyanotoxin is linked to the *nda* synthetase gene, a polyketide synthase (PKS) and nonribosomal peptide synthetase (NRPS) and biosynthesized by *Nodularia spumigena* NSOR10 cyanobacteria [4]. The review studies carried out by Pearson et al. [4] and Sinha [5] showed that cyanotoxin cylindrospermopsin is linked to the genes *aoa* or *cyr* (*A*–*O*) and is now known to be biosynthesized by a number of cyanobacteria genera, such as *Cylindrospermopsis* and *Umezakia natans* in Japan; *Aphanizomenon ovalisporum* in Israel, Australia, USA and Spain; *Anabaena bergii* in Australia; *Raphidiopsis raciborskii* in Thailand, China and Australia; *Raphidiopsis curvata* in China; *Aphanizomenon flos-aquae* in Germany; *Anabaena lapponica* in Finland; *Lyngbya wollei* in Australia; *Aphanizomenon gracile* in Germany; *Oscillatoria* sp. in the USA *Aphanizomenon* sp. in Germany; and *Raphidiopsis mediterranea*, *Dolichospermum mendotae* and *Chrysosporum ovalisporum* in Turkey.

The emergence of toxic cyanobacteria species during a bloom period is linked to environmental factors such as light, nutrient enrichment or nutrient depletion, and the presence or non-presence of predators [4]. Eutrophication, a build-up of organic matter produced by phototrophs, such as cyanobacteria [6,7], is often seen as algal blooms and driven by inputs of nitrogen and phosphorus. Cyanobacteria blooms are a major concern worldwide due to the production of cyanotoxins which are harmful to humans [8]. Cyanobacteria tend to proliferate during the summer when concentrations of total phosphorus fall to 100–1000 µg/L [9]. A variety of hypotheses explain why cyanobacteria blooms are becoming increasingly prevalent [10,11,12]. The most common hypotheses focus on nutrient conditions [10,11,13,14,15,16,17] and nutrient cycling [18] within a water body, as well as aspects of cyanobacteria cell physiology, such as their ability to migrate vertically within the water column, fix atmospheric nitrogen and to produce cyanotoxins [19,20,21,22].

Cyanobacterial blooms are often associated with eutrophic conditions [23,24,25]. Various studies have documented the relationship between nitrogen and phosphorus concentrations, speciation and stoichiometry, and cyanobacteria occurrence [10,13]. A recent study reported that *Microcystis* growth response increases in relation to nitrogen over phosphorus [26]. The same study [26] also reported that the growth response of toxic *Microcystis* to nitrogen was greater than non-toxic strains. Some species of cyanobacteria are known for their ability to fix nitrogen and thus giving them high chances of producing cyanotoxins [27]. Other studies have shown that microcystin toxicity is also influenced by changes in pH, temperature and light intensity [28,29,30]. A study conducted by Beversdorf et al., [27] indicated that some of the non-nitrogen fixing cyanobacteria may produce toxins because of nitrogen stress events.

However, a review of the available literature shows that there is limited information on the occurrence of toxic and non-toxic cyanobacteria species in relationship with river basin sediments on the African continent [31]. Thus, the main objectives of the study were to: (1) assess the physical-chemical characteristics of river sediments and how these contribute to the resurgence and growth of cyanobacteria species should ideal river flow conditions return; (2) use the FlowCam and molecular techniques to identify toxic and non-toxic cyanobacteria genes in the river sediments; and (3) to use the 16S rRNA in identifying the cyanobacteria species and explore relationships among the cyanobacteria species in the river sediments.

## 2. Results

### 2.1. The Physical-Chemical Characteristics of the River Sediments

**Figure 1:**
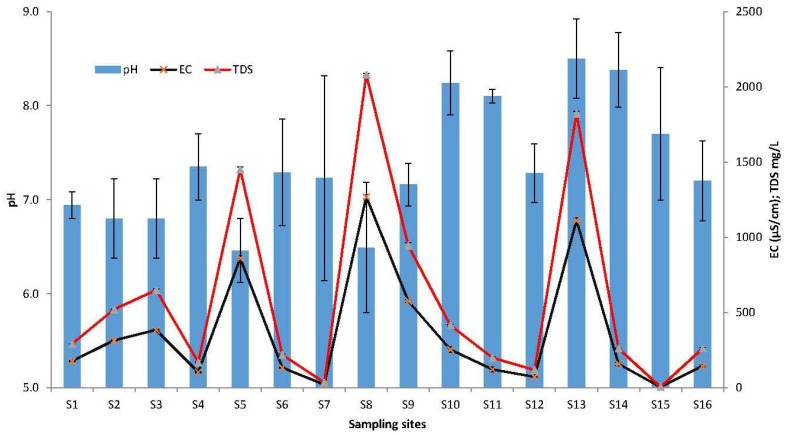
Average values of the physical characteristics of the river sediments of the 18 sampling sites. Whiskers reflect standard error. EC: electrical conductivity; TDS: total dissolved solids.

**Figure 2:**
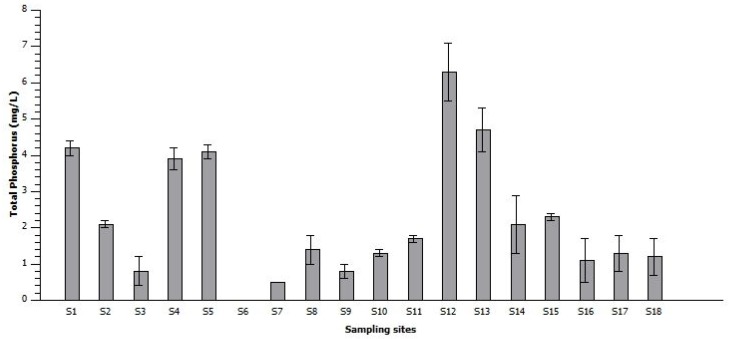
Average values of total phosphorus in the river sediments of the 18 sampling sites. Whiskers reflect standard error.

The physical characteristics of the river sediments drawn from the different tributaries of the Limpopo River basin and the Limpopo River itself showed considerable variation (Figure 1). The electrical conductivity (EC) and total dissolved solids (TDS) values in the river sediments ranged between 21.2 and 1269 μS cm^−1^ throughout the sampling sites. The pH values were between 6.4 and 8.5, while the total phosphorus concentration values in the river sediments ranged from 0.5 mg/L to 6.3 mg/L (Figure 2). The highest total phosphorus value was recorded for the sediments from the Nzhelele River (S12) near the Mphephu Resort and downstream of the Siloam oxidation ponds. The second highest phosphorus concentrations were measured at the Shashe River (S13), with the total phosphorus measuring 1.2 ± 0.5 mg/L at sample point S18.

**Figure 3:**
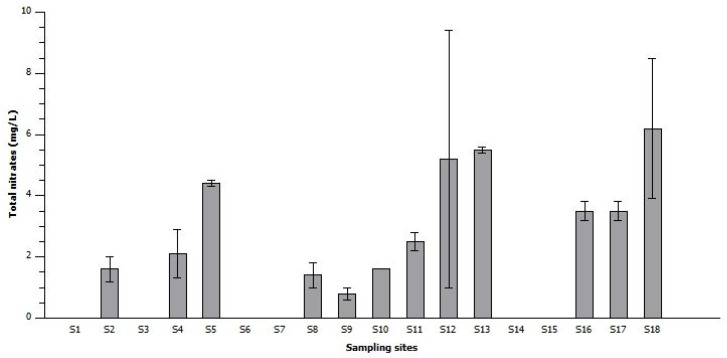
Average values of total nitrogen in the river sediments. Whiskers reflect standard error.

Finally, the nitrogen concentration values in the river sediments ranged from 1.5 mg/L to 6.5 mg/L (Figure 3). The highest concentrations were recorded for the sample from the Nzhelele River (S12) near the Mphephu Resort and downstream of the Siloam Hospital oxidation ponds, while the total nitrogen at site S18 was 6.25 mg/L.

### 2.2. The Presence of Cyanobacteria in the River Sediments

**Figure 4:**
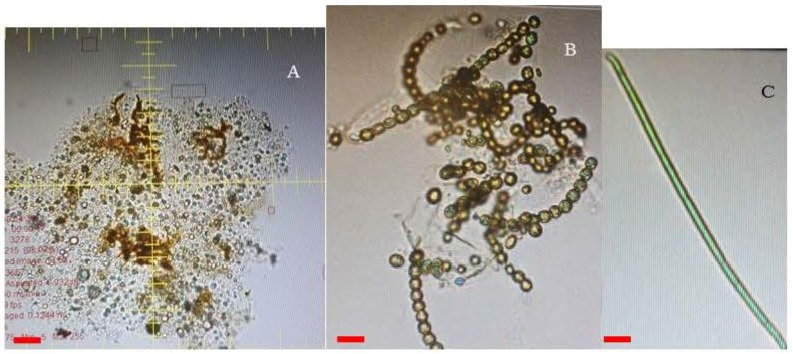
The (**A**) *Microcystis*, (**B**) *Anabaena* and (**C**) *Oscillatoria* species in the river sediments. Red scale bar = 20 µm.

Table 1 shows the presence of toxic and non-toxic cyanobacteria species that were detected in the Limpopo River basin. The dominant cyanobacteria observed is the filamentous *Leptolyngbya* species, followed by the Synechocystis species (non-toxic and toxic strains), toxigenic *Microcystis* species, and toxigenic Raphidiopsis raciborskii species. The FlowCam showed the presence of the different cyanobacteria species in the Limpopo River basin, as shown in Table 1 and Figure 4. The dominant cyanobacteria species identified from the samples were the *Microcystis* species, followed by the Raphidiopsis raciborskii, Calothrix, Phormidium and Planktothrix species. Finally, no cyanobacteria species were detected in the Mokolo River (S7).

### 2.3. PCR Analysis of the 16S rRNA Gene

**Figure 5:**
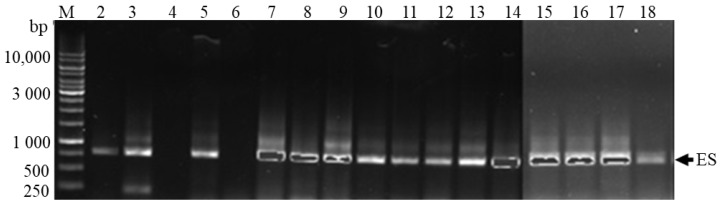
PCR amplification using 27F and 740R primer pair for 16S rRNA gene. ES (estimated fragments); M (Standard Marker), 2–18 Sample numbers. Lane 2 = Notwane River; 3 = Sand River upstream; 4 = Mogalakwena River; 5 = Mawoni River; 6 = Lephalale River; 7 = Mokolo River; 8 = Crocodile River downstream of Hartbeespoort Dam; 9 = Nzhelele River downstream; 10 = Sand River downstream; 11 = Crocodile River downstream (near the bridge on road D1235); 12 = Nzhelele River upstream; 13 = Mzingwane River; 14 = Shashe River; 15 = Limpopo River (next to Thuli Coalmine); 16 = Limpopo River (abstraction point at 0.0 m); 17 = Limpopo River (abstraction point at 1.0 m); 18 = Limpopo River (abstraction point at 1.68 m).

Multiple fragments were obtained for each sample by sequencing with both forward and reverse primers, and these samples were edited and assembled using the Staden package [32]. All assembled sequences were aligned in BioEdit v7.0.9 [33]. However, the sample collected from the Limpopo River (S1) was not shown in Figure 5, since it was used as the test sample. It was also noted that the amplified fragment from the test sample only produced 100 bp, while around 650 bp was expected. Other samples, such as the samples Limpopo River (S15), Limpopo River (S17) and Musina borehole abstraction point (S16), did amplify, but failed to assemble in the Staden package [32]. The assembled sequences were run on the BLAST algorithm [34] to identify closely similar sequences already deposited in GenBank via NCBI, and the outcomes are shown in Table 2.

It must be understood from the BLAST algorithm [34] that more than 98% similarity obtained matches the sample to the correct species, more than 90% similarity obtained matches the sample to the correct genus, while more than 80% similarity obtained matches the sample at the Family level. The PCR products that were separated by gel electrophoresis are shown in Figure 5. The presence of the different bands indicated a positive amplification, whereas a blank sample indicated a negative amplification. The blank samples where repeated several times and failed to amplify. Almost all of the samples showed positive amplification, which confirmed the presence of cyanobacterial DNA in the samples. The two samples which showed no amplification were drawn from the Mogalakwena (S4) and Lephalale Rivers (S6). The BLAST algorithm [34] showed that more than 98% similarity obtained matches the sample to the correct species, more than 90% similarity obtained matches the sample to the correct genus, while more than 80% similarity obtained matches the sample to the correct family.

BLAST data from the samples drawn from the Mokolo River (S7), Crocodile River downstream of Hartbeespoort Dam (S8) and the Shashe River (S14) did not identify the cyanobacteria up to the species level. However, the cyanobacteria from the samples drawn from the Notwane River (S2), Sand River upstream (S3), Mawoni River (S5), Nzhelele River downstream (S9), Sand river downstream and the Limpopo River (S18) (abstraction point at 1.68 m) were identified up to genus level. Lastly, the cyanobacteria from the sampled Limpopo River (S16) (abstraction point at 0.0 m) was identified up to the family level. Furthermore, the samples from Crocodile River downstream (S11) (near bridge on road D1235), Nzhelele River upstream (S12) and Mzingwane River (S13) showed similarities and there were no families that could be detected for these samples.

### 2.4. Detection of Genes Involved in Toxin Production

**Figure 6:**
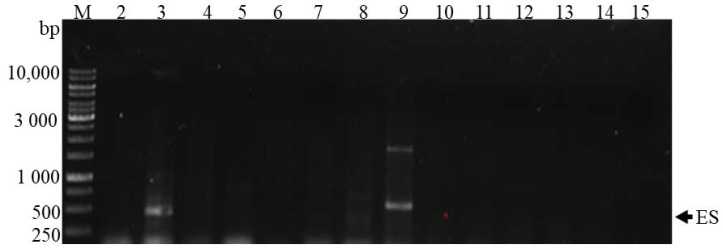
PCR products using PKS primers for cylindrospermopsin polyketide synthetase gene. ES (estimated fragment); M (Standard Marker), 2–18 Samples number. Lane 2 = Notwane River; 3 = Sand River upstream; 4 = Mogalakwena River; 5 = Mawoni River; 6 = Lephalale River; 7 = Mokolo River; 8 = Crocodile River downstream of Hartbeespoort Dam; 9 = Nzhelele River downstream; 10 = Sand River downstream; 11 = Crocodile River downstream (near the bridge on road D1235); 12 = Nzhelele River upstream; 13 = Mzingwane River; 14 = Shashe River; 15 = Limpopo River (next to Thuli Coal Mine).

The detection of cyanotoxins was done through detecting genes for the proteins that make toxins. This was achieved with PCR by amplification of microcystin/nodularin synthetase using the HEP primer pairs and cylindrospermopsin polyketide synthetase genes using a PKS primer pair. It must be noted that the detection of the genes involved in the biosynthesis of toxins does not confirm the production of the toxins in the field. The *mcyA-C* primer pair and M13 and M14 primer pair were also used to determine the presence of the genes that contain the proteins for toxins production. However, the genes were not detected, as there was no amplification in most of the samples of any of the genes associated with the proteins that produce toxins. Nevertheless, a few samples, such as the Sand River (S3) upstream and Nzhelele River (S9) downstream (Figure 6), showed the amplification of the cylindrospermopsin polyketide synthetase gene. This confirmed the presences of cyanotoxin, cylindrospermopsin in the sediment samples and was attributed to the cyanobacteria species, *Raphidiopsis raciborskii* (Table 3).

**Figure 7:**
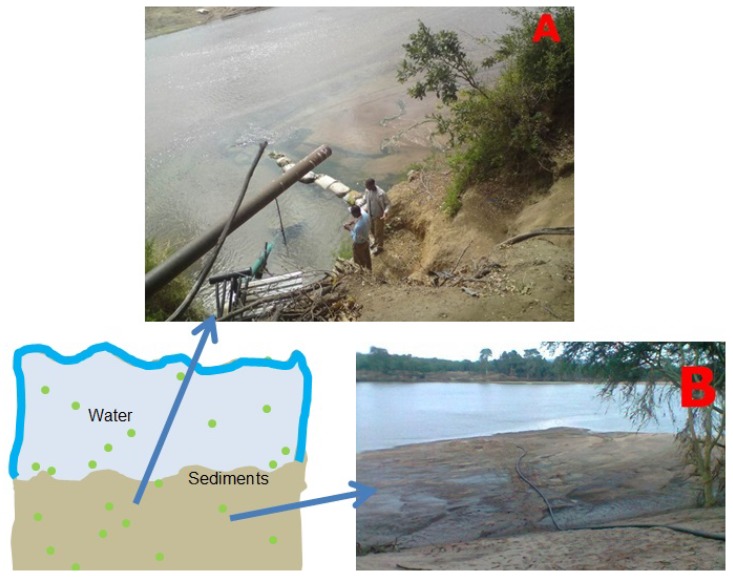
A scenario involving boreholes drilled inside the Limpopo river channel and contamination with cyanobacteria (green dots) cysts and akinetes for (**A**) irrigation farmers & (**B**) water utility raw water supply for human consumption.

The HEP primer pair produced two positive results for samples from Crocodile River (S8) and Mzingwane River (S13). Both positive results were attributed to the presence of toxigenic *Microcystis* sp. (Table 3). The latter is an important finding, since water supplies from the Limpopo River basin are used by water utilities for drinking water supplies, and by commercial and subsistence irrigation farmers for the production of food crops and livestock watering (Figure 7).

### 2.5. Phylogenetic Relationship

**Figure 8:**
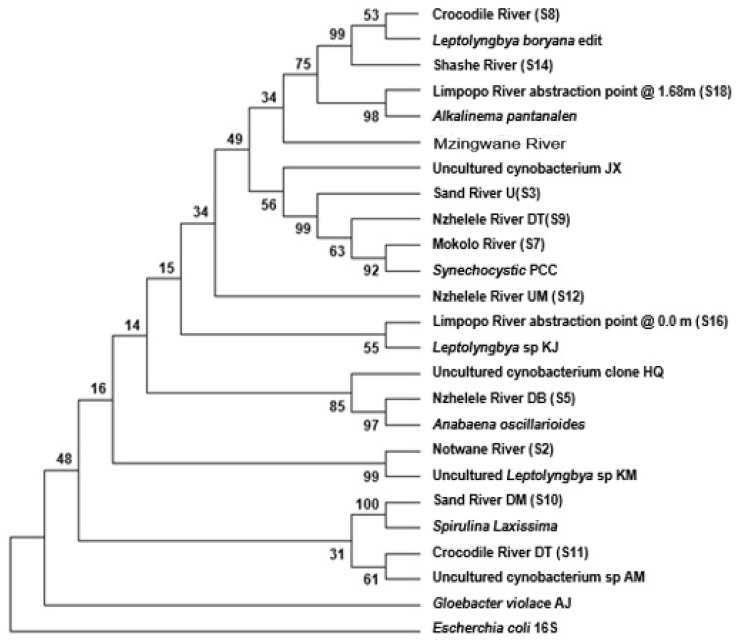
The evolutionary history was inferred using the Neighbor-Joining method. U: upstream; UM: Upstream; DT: Downstream; DB: Downstream; DM: downstream; PCC: Pasteur Culture Collection of Cyanobacteria

The relationship between the samples and their most similar species, as noted from the BLAST search, was confirmed by the phylogenetic tree, and the relationships between some cyanobacteria species from different samples (Figure 8). The first confirmation was on the similarity of samples from the Crocodile River (S8) downstream Hartbeespoort Dam and the Shashe River (S14) to *Leptolyngbya* boryana with 99% bootstrap confidence. Secondly the similarity of the Musina borehole extraction (S16) sample to Alkalinema pantanalense, with 98% bootstrap, and the similarity of the samples from Sand River (S3) upstream. Another similarity was evident in the Nzhelele River (S9) downstream near Tshipise and Mokolo River to Synechocystis sp. PCC 6803. The other similarity was Mawoni River (S5) downstream of Makhado oxidation pond to *Leptolyngbya* sp. with 97% bootstrap confidence, with the Notwane River (S2) to uncultured *Leptolyngbya* sp. with 99% bootstrap confidence, and lastly the Sand River (S10) downstream to the filamentous cyanobacterial species Spirulina laxissima with 100% bootstrap confidence.

**Figure 9:**
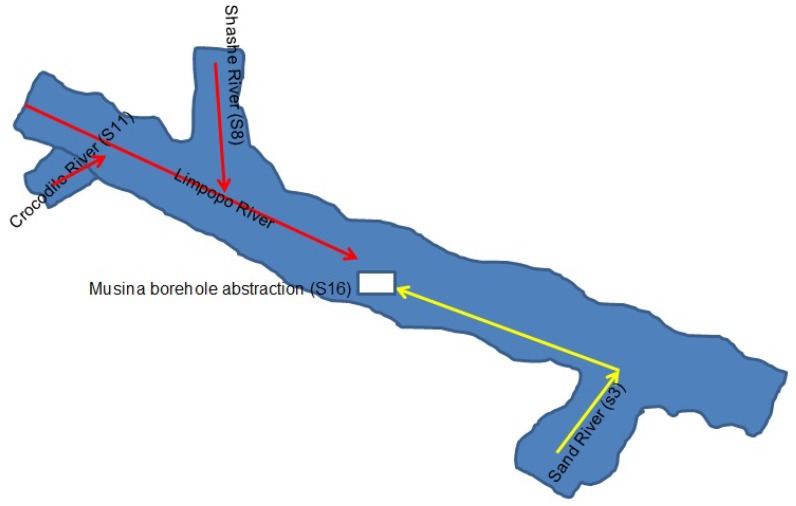
A scenario involving the movement of cyanobacteria species during water flows in the Limpopo River (red arrow) towards the Musina abstraction borehole (White Square). The possible upstream movement (yellow arrow) from the Sand River (S3) to the Musina borehole (S16) may involve cyanobacteria ‘hitching a ride’ on aquatic animals such as fish and crocodiles.

However, the detection of cyanobacteria at the Musina borehole extraction (S16) and the Sand River (S3) may suggest that there were aquatic animals such as fish, dispersal or transportation of cyanobacterial cells from the entrance (mouth) of Sand River towards the Musina abstraction point (Figure 9). In simple terms, there was an upstream transport of cyanobacteria species that was facilitated by aquatic animals, but this requires further investigation. The other matches from the BLAST search include the Musina Borehole extraction point (S16) to *Leptolyngbya* sp., as well as the Crocodile River (S11) near bridge on road D1235 and upstream of Thabazimbi town to uncultured Cyanobacteria clone. However, the latter bootstrap confidence levels were between 55 and 61%, respectively. It was evident from the data that a divergence matrix can be used to verify the truth of both the BLAST search and phylogenetic tree. The divergence matrix confirmed that cyanobacteria from the Crocodile River (S8) downstream of Hartbeespoort Dam and from Shashe River (S14) were the same *Leptolyngbya* boryana species, since they both showed at least 98% similarity to this species.

Thus, the current study indicates that there is DNA evidence to suggest a similarity between the cyanobacteria at the Musina abstract point and that from the Crocodile River system. The possibility thereof lies in the fact that the Musina abstraction point was downstream from the Crocodile River, which flows into the Limpopo River (Figure 7). However, samples from the Nzhelele River upstream near the Mphephu Resort and Mzingwane River (Zimbabwe) did not match. Nevertheless, the relationship between the cyanobacteria species from specific locations were identified by a Divergence Matrix (Table 4). In addition, the same species were detected by the difference co-efficient of 0.00, whereas completely unrelated species were detected by the co-efficient of 1.00.

The cyanobacteria species from the Crocodile River (S8) were the same species as the cyanobacteria from the sampled Shashe River (S14), since they had less than 1% difference (0.006). This may have been expected, since the Shashe River is downstream of the Crocodile River (Figure 9). The cyanobacteria species from the Mokolo River (S7) and the Nzhelele River (S9) share undetectable differences. However, a comparison shows that there was a difference between the cyanobacteria species from the Notwane (S2) and Mawoni Rivers (S5). The cyanobacteria which differed the most from the other species were the cyanobacteria species from the Notwane River (S2) and Limpopo River (S16). The latter sampling sites’ species also differed from each other, with 28%, while their comparison co-efficient range from 0.17 to 0.28. Furthermore, the Nzhelele River upstream (S12) and Limpopo River (S16) species differed from each other with 28%, while their comparison co-efficient ranged between 0.312 to 0.492, which was the highest for all species.

The first observation was that Uncultured Cyanobacterium clone HQ189039.1 could not be used for the phylogenetic tree because of its length (about 480 bp). This is because the t complete deletion option of gaps and missing information in MEGA 7 [35] was used. The second observation was that two outgroup sequences had been used in phylogenetic alignment.

## 3. Discussion

The physical chemical data generated in the current study shows that there were large variations in sediment EC between the different sampling sites while the sediment temperature was ≥22 °C during all the sampling trips. High temperatures arising from climate change have been reported as an important factor in the global expansion of harmful algal bloom worldwide [36]. Rising temperature exceeding 20 °C can promote the growth rate of cyanobacteria, whereas the growth rate other freshwater eukaryotic phytoplankton decreases, which is regarded as a competitive advantage for cyanobacteria [37]. A study by O’Neil et al. [9] reported that higher temperatures promote the dominance of cyanobacteria and favor the production of microcystins, as well as resulting in an increase in their concentration.

The high pH value measured during the current study may have a competitive advantage for many cyanobacteria, because of their strong carbon-concentrating abilities compared to eukaryotic phytoplankton species [38]. A laboratory experiment carried out by Jahnichen et al. [39] on *Microcystis aeruginosa* showed that microcystin production started when pH exceeded 8.4, thus indicating a lack of free carbon dioxide (CO_2_).

The increased input of nutrients into the surface water is the main factor responsible for massive proliferations of cyanobacteria in fresh water, brackish and coastal marine ecosystems. However, phosphorus and nitrogen nutrients in high levels lead to accelerated growth of cyanobacteria [40,41].

Thus, the higher concentration of phosphorus measured in the current study downstream of the Siloam oxidation ponds may be due to the discharge of sewage effluent [42]. The low concentration of phosphorus measured in the Lephalale River (S6) is possibly related to less anthropogenic land use activities upstream of this sample site [43]. Phosphorus has been implicated more widely than nitrogen as a limiting nutrient of phytoplankton and cyanobacteria in freshwater systems [44]. A minimum amount of phosphorus entering or becoming soluble in a water body can trigger a significant algal bloom [45]. The impact of excess phosphorus on receiving rivers or streams is evident from the green coloration of surface water owing to the presence of phytoplankton or cyanobacteria. The Limpopo River (S1) receives inflows from both the Notwane and Crocodile Rivers, and these contribute significantly to the phosphorus loading of the Limpopo River. Furthermore, the main source of phosphorus in the Notwane River (S2) is the municipal discharge from the Glen Valley sewage plant and agricultural runoff from irrigated farms and livestock ranching in the river’s catchment [23,46]. The Crocodile River receives sewage effluent from upstream catchment land use activities such as the discharge of sewage effluent into tributaries of the Crocodile River, discharge into Crocodile River itself and agricultural runoff [47,48]. The Sand River (S3) receives municipal nutrient discharge from the Polokwane sewage plants and rainwater runoff that would contain fertilizer from agricultural activities in the river sub catchment [49]. The sample point on the Mogalakwena River (S4) was downstream of the Mokopane, Modimolle and Mookgophong towns’ sewage plants, golf courses, game farming, livestock farming and irrigated farmlands [50]. After the town of Mokopane, the Nyl River is renamed to the Mogalakwena River. The Mzingwane River (S13) receives municipal discharge from the Filabusi, Gwanda and West Nicholson sewage plants and agricultural runoff from irrigated farms and livestock ranching in these areas [51], and this may be attributed to sewage plants upstream in Francistown and agricultural runoff from irrigated farms and livestock ranching [48,52]. These rivers are part of the Limpopo River’s tributaries and contribute to the successive loading of phosphorus in the Limpopo River (S15–S16) [53].

The highest value of nitrogen was recorded in the Nzhelele River (S12) near the Mphephu Resort and downstream of Siloam hospital oxidation ponds. The reason for the detection of these high nitrogen values is possibly related to the discharge of sewage effluent from the Siloam hospital [42]. Filamentous cyanobacteria can obtain nitrogen by fixing the atmospheric nitrogen gas and converting it to nitrate for their growth [54]. Nitrogen is a common gas (79%) that is found in the atmosphere. Thus, cyanobacteria genera such as *Anabaena* are able to utilize atmospheric nitrogen in addition to nitrate originating from the river sediments [54,55]. The other sample sites with nitrates in excess of 2 mg/L are Sand River (S4), Mawoni River (S5), Crocodile River (S11), Mzingwane River (S13), and Limpopo River (S16 to S18). All these tributaries have one in common source of pollution upstream, namely, a municipal sewage plant, and are also surrounded by farmland where commercial irrigation farming is practiced, as in the case of the Crocodile, Notwane, Shashe, Mzingwane and Sand rivers. Subsistence agriculture is practiced in the case of the Mawoni and Mzingwane rivers [46,48,49,50,51,52]. The Crocodile River also receives inflows from eutrophic Hartbeespoort Dam [47]. The Limpopo River (S16) is downstream of all the sample points, and this shows the cumulative discharge of nitrates originating from the tributaries, causing an increase in concentration of nitrogen. The Musina local municipality has drilled 8 boreholes in the Limpopo river bed, and most of these boreholes are located close to S16, and thus there is a possibility of cyanotoxin contamination of the borehole water [56]. Thus, further research is required to determine if there is cyanotoxin contamination of borehole water.

Botha and Oberholster [57] performed a survey of South African freshwater bodies between 2004 and 2007, using RT-PCR and PCR technology to distinguish toxic and non-toxic *Microcystis* strains bearing *mcy* genes, which correlate with their ability to synthesize the cyanotoxin microcystin. The study revealed that 99% of South Africa’s major impoundments contained toxicogenic strains of *Microcystis*. The study by Su et al. [58] in the Shanzi impoundment, China showed that the sediments were the source of cyanobacteria inoculum. This implies that the cyanobacteria flocculates in the sediments during periods of adverse environmental conditions, such as cessation in river flows. These cyanobacteria cysts or spores then reactivate during periods of river flow. The cyanobacteria cysts and spores are related to the summer environmental conditions in the Limpopo river basin, where the majority of tributaries are perennial and river flows commence during the period of summer rainfall. The river flows disturb the sediments, thus bringing into the water column the cyanobacteria cysts or spores [58]. The source of nutrients in the Limpopo river basin may be attributed directly to sewage discharge of municipal waste water plants such as Glen Valley and Mahalpye on the Botswana side, and on the South Africa side, the western and northern parts of the city of Johannesburg to the town of Musina and indirectly to the agricultural practices of fertilizer application and animal waste [46,48,49,50,51,52].

**Figure 10:**
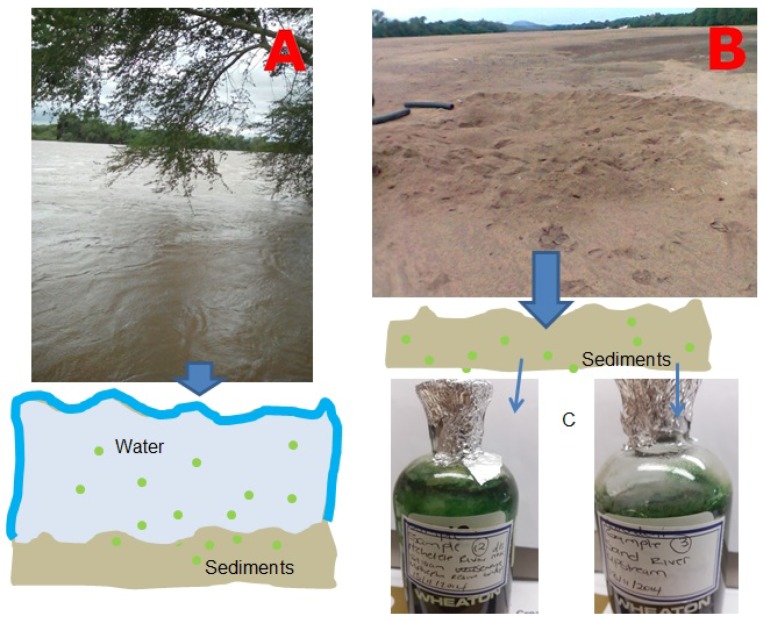
Scenario involving sedimentation of cyanobacteria (green dots) cysts and akinetes (**A**) during flood and flow conditions in Limpopo River and (**B**) during non-flow (DRY) conditions in the Limpopo River and (**C**) growth of cyanobacteria under continuous lighting and provision of BG medium at room temperature.

Cyanobacteria undergo distinct developmental stages [59]. For example, they differentiate into resting cells, spores, akinetes and cysts which represent a survival strategy under unfavorable environmental conditions [55,60]. Under favorable conditions, the cell will germinate again [61]. The ability of cyanobacteria to adapt to adverse dry periods allows them to inhabit the river sediments, as shown by studies by Perez at al. [60], Kim et al. [55] and this study (Figure 10). The study of Kim et al. [55] further illustrated the viable nature of cysts and akinetes in providing the next inoculum of *Microcystis, Anabaena, Aphanizomenon* and *Oscillatoria* is Bukhan, Namhan Rivers and Lake Paldang and Kyeongan stream, in South Korea.

As expected, the toxigenic *Microcystis* species was found in the Crocodile River, downstream of the Hartbeespoort Dam, a eutrophic water impoundment known for the regular occurrence of *Microcystis* dominated harmful algal blooms [40]. However, our two toxigenic *Microcystis* strains were different from the seventeen toxigenic *Microcystis* strains studied by Mbukwa et al. [24] from the Hartbeespoort Dam. The differences may be explained by the different use of *mcy* primers in identifying the genes expressing toxicity and differences in experimental approach. Our study on the *mcyA-Cd* primer did not amplify, whereas Mbukwa et al. [24] reported the amplification of the *mcyA-Cd* genes, showing seventeen toxigenic *Microcystis* strains. However, during our study, the *mcyE* genes were positively expressed with the HEP primer and did amplify, but the outcomes were also different from the toxigenic *Microcystis* strains studies by Mbukwa et al. [24]. During our study, the total genomic DNA was not extracted directly from the sediments, but from cyanobacteria that was cultured in the laboratory. Laboratory culture conditions have been known to alter the toxicity of *Microcystis* species, as shown by the study of Scherer et al. [62]. In the latter study, the authors mimicked a temperature increase of 10 °C. Under these increased temperature conditions, *Microcystis* was able to express *mcyB* gene related to production of toxicity instead of the *mcyD* gene. This may imply the biodiversity of toxigenic *Microcystis* strains in Hartbeespoort Dam and the Crocodile River and the Limpopo River basin. 

Mbukwa et al. [24] used DNA molecular techniques to identify the two species of *Microcystis* as *M. aeruginosa* (origins from Hartbeespoort Dam, South Africa) and *M. novacekii* (origins from Phakalane effluent, Gaborone, Botswana). The molecular techniques showed the presence of the *mcy* genes responsible for microcystin encoding, thus confirming that the two *Microcystis* species did have the potential to produce toxins. The Phakalane pond effluent is discharged into the Notwane River, a tributary of the Limpopo [23]. An earlier study by Basima [51] upstream of the Mzingwane S13 sample point showed the abundance of cyanobacteria genera dominated by *Microcystis* species followed by *Anabaena* and *Nostoc* species in water impoundments situated inside the Mzingwane River. In the lower Limpopo River, situated in Mozambique, at the Chokwe irrigation scheme, which receives irrigation waters from Maccaretane Dam, Pedro et al. [25] reported the presence of *Microcystis* species and microcystin-LR concentrations of 0.68 ppb. The latter concentrations were linked to the presence of the *mcyB* and *mcyA* genes in collected water samples. Mikalsen et al. [63] identified eleven *Microcystis* species containing different variants of the *mcyABC* (toxic species), and seven *Microcystis* species that lacked the *mcyABC* gene (non-toxic species). A study by Davies et al. [64] on four temperate lakes in the northwest of the USA showed that the increase in water temperature contributed to an increase in toxic *Microcystis* species (possessing the *mcyD* gene). Yamamoto [65] and Oberholster et al. [66] have shown that the *Microcystis* species adopt survival strategies to mitigate harsh external environments such as reduced river flow, a major characteristic of the Limpopo River, by sinking into the sediments.

The Limpopo river basin is characterized by extreme weather events such as heatwaves, floods and drought [67]. Could the latter, including extreme heatwaves, possibly have contributed to toxic or non-toxic *Microcystis* species? The presence of microcystins in the rivers may constitute a health risk, especially for the communities that may be in contact or drink the polluted water without any form of treatment or suitable treatment that is able to remove the toxins in the water. The convectional method for water treatment is not convenient for the removal of microcystins in water [68]. Drinking water treatment processes might trigger the release of hepatoxin into drinking water by disrupting the trichomes of cyanobacteria [69]. Thus, the presence of cyanotoxins can also poison the livestock and game animals (wildlife) in transfrontier parks such as Kruger National Park, Gona-re-zhou National Park and Mapungubwe National Park [70]. Microcystins have already been implicated in the death of wildlife in the Kruger National Park [71]. Cyanotoxins have been implicated in the negative growth (stunting) of plants, and this may have serious repercussions for irrigation farmers [72].

The evolutionary tree constructed could not be used for phylogenetic purposes because of two important reasons: (1) the number of samples used for PCR per river site was not enough to make a conclusive argument; and (2) the cyanobacteria were the expected products which needed to be identified. Hence the tree was used to verify the identification as done by BLAST search; however, the phylogenetic relationship was basically done by divergence matrix, and combined discussion followed the divergence matrix.

## 4. Conclusions

Many countries in Africa have reported cases of intoxication and death of animals that may have been caused by cyanobacterial toxins. Monitoring and or reducing the nutrient loads into the river system will decrease the threat of cyanobacteria blooms to human and animal health. The results obtained in the current study indicated the presence of toxic and non-toxic cyanobacteria species in the bottom sediments of the Limpopo River and its tributaries. Molecular tools were used in the present study to determine non-toxic and toxic cyanobacteria based on genes that produce proteins related to cyanotoxins. The presence of nutrients, phosphates and nitrates in the river sediments did stimulate the growth of the cyanobacteria during summer river flow periods. Furthermore, the expression of genes that have the potential to produce toxins, for example cylindrospermopsin and microcystin/nodularin in the river sediments indicate a potential risk to the environment and human health. The cyanotoxins are harmful to humans who consume the water originating from boreholes located inside the Limpopo River basin or drilled along the Limpopo River basin. Secondly, the water supplies from the Limpopo River basin are used by commercial and subsistence irrigation farmers for growing food crops and livestock watering. Thus, presence of cyanotoxins can also adversely affect the livestock and game animals (wildlife) in transfrontier parks. Furthermore, cyanotoxins have been implicated in the negative growth (stunting) of plants, and this may have serious repercussions for the irrigation farmers in this region.

## 5. Future Research Work

Further studies are required to determine the level and types of cyanotoxins in the Limpopo river basin, since water resources are used for a variety of purposes, such as human consumption, irrigation, livestock and wildlife watering and impact if any on aquatic biodiversity.

## 6. Materials and Methods

### 6.1. The Study Area

**Figure 11:**
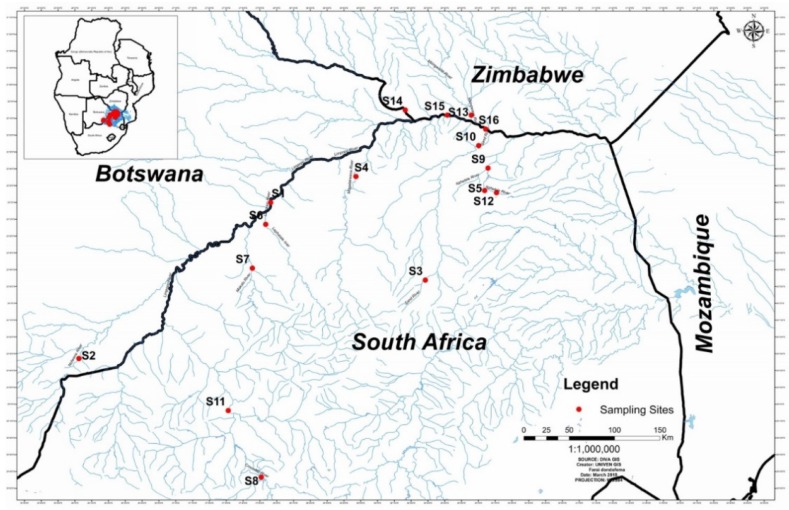
The location of sediment sample sites on some of the tributaries of the Limpopo River.

The study area is the Limpopo River and its major tributaries (Figure 11). The Limpopo River basin consists of four countries: Botswana, South Africa, Zimbabwe and Mozambique [73].

The Limpopo River basin is an arid to semi-arid region where water is of strategic importance to development. Water has a potential limiting effect on all future development in the region. The Limpopo River basin is home to almost 14 million people in four riparian states [74].

### 6.2. Sampling Sites and Sampling Methods

Sampling sites were selected with the following in mind: (a) accessibility, biotype, e.g., sandy bottom sediment; (b) canopy cover and depth, and (c) river sites receiving inflows of municipal sewage discharges. The 18 grab river sediment samples were collected in October and November 2014. The river sediment samples (~500 g) were collected in sterile glass containers from rivers and tributaries of the Limpopo River (Table 5). The use of river sediments was chosen because most suspended material, including cyanobacteria spores and cysts, settles at the river bottom, where they become part of the sediments in river systems.

### 6.3. Physical-Chemical Measurements

In the laboratory, the physical measurement of pH, Total dissolved solids (TDS) and electric conductivity (EC) was carried out using Portable pH meter Crison MM40 (Crison Instruments SA, Alella, Spain) on the river sediments. It was first calibrated as per the manufacturer’s guidelines. The pH, TDS and EC of the sediments were determined by the method of Islam et al. [75], in which 50 g of sediment was mixed with 50 mL of distilled water in a 100 mL beaker to produce a ratio of 1:1. The mixture was stirred with a stirring rod to homogenize the mixture and was then left for 30 min to settle. EC, pH and TDS were then measured by inserting the electrodes in the soil solution and readings were taken.

### 6.4. Nutrient Analysis

The air-dried sediments were subjected to nutrient analysis, and this involved determining Total Phosphate (TP) and Total Nitrogen (TN). The analyses were done in duplicates and the aliquot of all digested samples was analyzed with Merck Spectroquant^®^ Pharo 100 spectrophotometer with a wavelength of 320–1100 nm purchased from Merck (Darmstadt, Germany).

#### 6.4.1. Total Phosphorus Analysis

Total phosphorus was determined by using the perchloric acid digestion method as described by American Public Health Association (APHA) [76]: 2 g of air-dried sediment was acidified to methyl orange with concentrated HNO_3_, another 5 mL of concentrated HNO_3_ was added and evaporated on a hotplate until dense fumes appeared. 10 mL each of concentrated HNO_3_ and HClO_4_ was added and evaporated gently until dense white fumes of HClO_4_ appeared. The solution was then neutralized with 6N NaOH and made up to 100 mL with distilled water. Aliquots of the samples were then analyzed with spectrophotometer using phosphate cell test kit (Merck, Darmstadt, Germany).

#### 6.4.2. Total Nitrogen Analysis

Total nitrogen was determined per APHA [76] as ammonia: 1 g of each air-dried sediment sample was treated with 2 mL of sulphuric acid. The sample was heated on a hotplate for 2 h. Aliquots of 50 mL of deionized water were added to each sample. The sample was filtrated through No. 41 Whatman filter paper. The filtrate of each sample was made up to 250 mL with deionized water and 55 mL of 1 M sodium hydroxide solution. Aliquots of the samples were then analyzed with spectrophotometer using a nitrate cell test kit (Merck, Darmstadt, Germany).

### 6.5. Data Analysis

The physico-chemical and cyanotoxin measurements were conducted in duplicates, and the standard deviation and mean were calculated, using a Microsoft (MS) Excel 2010 spreadsheet for each sampling point. The graphs were plotted using MS Excel.

### 6.6. The Culture of Cyanobacteria Species in River Sediments

The modified BG11 medium was laboratory-prepared as per Gumbo et al. [77] for cyanobacteria culturing. The 200 mL sterile modified BG11 medium was transferred to sterile 250 mL laboratory jars under sterile conditions and then 200 g of river sediments was added. A total of 18 laboratory jars were incubated for 30 days under continuous light (1100 lux) fluorescent lamps at room temperature. The harvested cyanobacteria cells were subsequently used for identification and molecular characterization.

### 6.7. The Identification of Cyanobacterial Species Using the FlowCam

The harvested cyanobacteria cells were used to identify cyanobacterial species present in the samples, a bench top FlowCam (Model vs. IV) was used. In the FlowCam system, the sample is drawn into the flow chamber by a pump. Using the laser in trigger mode, the photomultiplier and scatter detector monitor the fluorescence and light scatter of the passing particles. When the particles passing through the laser fan have sufficient fluorescence values and/or scatter, the camera is triggered to take an image of the field of view. The fluorescence values were then saved by the Visual Spreadsheet. The computer, digital signal processor, and trigger circuitry work together to initiate, retrieve and process images of the field of view. Groups of pixels that represented the particles were then segmented out of each raw image and saved as a separate collage image. The image was then captured and compared to the image of cyanobacteria as per the procedure of van Vuuren et al. [78].

### 6.8. The Identification of Cyanobacterial Species Using Molecular Characterization

The cyanobacteria cells were harvested and used for molecular characterization as per the following procedures, as outlined below:

#### 6.8.1. DNA Extraction and Purification

Samples were freeze-dried and stored at −20 °C for DNA extraction. Total genomic DNA was extracted using the ZR-Duet^TM^ DNA/RNA Miniprep DNA extraction kit from Inqaba Biotech Laboratories South Africa (Pretoria, South Africa). Sample preparation and DNA extraction was carried out following the protocol supplied by the manufacturer.

#### 6.8.2. Detection and Amplification of 16S rRNA by Polymerase Chain Reaction

The PCR method was performed for detection and amplification of 16S rRNA as described briefly by Frazao et al. [3]. The PCR amplification of the cyanobacteria 16S rRNA gene was determined using set of primers 27F/809R (Table 2). Thermal cycling conditions were 1 cycle at 95 °C for 5 min, 35 cycles at 95 °C for 30 s, 55.4 °C for 30 s and 72 °C for 60 s and 1 cycle at 72 °C for 10 min. Reactions were carried out in a 50 μL reaction volume that consisted of 0.5 pmol of each primer (10 pM/μL), 25 μL of Dream Taq master mix (Inqaba Biotech), 19 μL sterile ultra-pure water and 5 μL of DNA sample.

#### 6.8.3. Toxin Gene Detection

The presence of cyanotoxins was determined by PCR using primers that were used for detection of genes involved in the production of nodularins (NOD), microcystins (MC) and cylindrospermopsin (CYN) (Table 6). The NOD gene cluster, *nda*, consists of nine open reading frames (*ndaA-I*) [79]. The MC gene cluster, *mcy*, comprises 10 genes in two transcribed operons, *mcyA-C* and *mcyD-J* [80]. The HEP primer pair was used for detection of genes involved in MC and NOD production.

These primers are responsible for sequencing the aminotransferase (AMT) domain, which is located on the modules *mcyE* and *ndaF* of the MC and NOD synthetase enzyme complexes, respectively [80,81]. Primers *mcyA-C* were used to detect the *mcyA*, *mcyB* and *mcyC* genes [82,83]. For detection of CYN production (*cyr*) genes, the polyketide synthase PKS M4 and M5 primers and the peptide synthetase M13 and M14 primers were used as designed by Schembri et al. [84], who demonstrated a direct link between the presence of the peptide synthetase and polyketide synthase genes and the ability of cyanobacteria to produce CYN.

The PCR reaction conditions that were used were those described for the amplification of the 16S rRNA gene [81]. Concerning the cycling conditions, for *mcyA-Cd* genes, the thermal cycling conditions were 1 cycle at 95 °C for 2 min, 35 cycles at 95 °C for 90 s, 56 °C for 30 s and 72 °C for 50 s and 1 cycle at 72 °C for 7 min. For HEP and CYN as genes, the thermal cycling conditions were as those for the amplification of the 16S rRNA with an exception for HEP gene annealing temperature of 58.15 °C for 30 s. Positive control was used.

##### Electrophoresis

PCR products were electrophoresed in 0.8% agarose gel by adding prepared 1.2 g of agarose powder into 150 mL 1X TAE buffer (48.4 g Tris, 11.4 mL Glacial acetic acid, 3.7 g EDTA disodium salt topped up to 1000 mL with deionized water). The mixture was heated until there was complete dissolution. Exactly 10 µL of ethidium bromide was added and mixed thoroughly. The mixture was transferred to the gel-casting tray with the comb already in position and allowed to solidify. The solidified gel was transferred to the running trays. The gel in the tray was covered with 1X TAE buffer. In the first well 3 µL 100 bp of the molecular weight marker was loaded and the samples were loaded from the second well onwards. The gel was run at 100 V and 250 mA for 60 min. The gel was viewed using the Gel doc (Biorad, Hercules, CA, USA) and the picture was taken.

#### 6.8.4. PCR Purification and Sequencing

PCR products were purified using the GeneJet Gel Extraction Kit Thermo Scientific (Pretoria, South Africa) under room temperature as per the protocol provided by the kit manufacturer. The purified DNA was stored at −20 °C. PCR products were sent for sequencing at Inqaba biotech laboratory (Pretoria, South Africa). Sequences were analyzed using the BLAST system (http://www.ncbi.nlm.nih.gov/BLAST/).

##### Primers

Primers used for PCR amplification were synthesized at Inqaba Biotech (Pretoria, South Africa). Details of primer sequences, their specific targets and amplicon sizes are summarized (Table 6) below.

#### 6.8.5. Phylogenetic Relationship

Additional sequences were downloaded in FASTA format from GenBank through NCBI and combined with assembled sequences. The evolutionary history was inferred using the Neighbor-Joining method [87]. The bootstrap consensus tree inferred from 1000 replicates [88] is taken to represent the evolutionary history of the taxa analyzed [88]. Branches corresponding to partitions reproduced in fewer than 50% bootstrap replicates are collapsed. The percentage of replicate trees in which the associated taxa clustered together in the bootstrap test (1000 replicates) is shown next to the branches [88]. The evolutionary distances were computed using the Kimura 2-parameter method [35] and are in the units of the number of base substitutions per site. The analysis involved 25 nucleotide sequences. Codon positions included were 1st + 2nd + 3rd + Noncoding. All positions containing gaps and missing data were eliminated. There were a total of 640 positions in the final dataset. Evolutionary analyses were conducted in MEGA7 [34].

#### 6.8.6. Divergence Matrix

PCR products for the 16S rRNA gene, identified on agarose gels, were selected for subsequent identification by sequencing (Inqaba Biotech, Pretoria, South Africa). The obtained sequenced data were used to conduct homology searches on GenBank using BLAST (http://blast.ncbi.nlm.nih.gov/blast.cgi) [89], and for further bioinformatic analyses to perform divergence matrix using BioEdit v7.0.9 [33]). Sequences were exported to and analyzed with the MEGA 7 package [34].

## Figures and Tables

**Table 1:** Summary of toxic and non-toxic cyanobacteria species in the Limpopo river basin.

Cyanobacteria Species/Sample Sites	S1	S2	S3	S4	S5	S6	S7	S8	S9	S10	S11	S12	S13	S14	S15	S16	S17	S18
*Aphanizomenon* sp.			+ *															
*Raphidiopsis curvata*			+ *															
*Microcystis aeruginosa*	+		+	+	+			++ *		+	+	+	++ *	+	+		+	
*Microcystis panniformis*								+ *										
*Synechocystis PCC 6803*			+ *				+		+									
*Cylindrospermopsis* sp.			+ *						+ *									
*Lyngbya* sp.	+																	
*Leptolyngbya* sp.		+														+		
*Leptolyngbya boryana*								+						+				
*Calothrix* sp.		+						+	++ *				+	+				
*Oscillatoria* sp.		+	+						++ *									
*Phormidium* sp.		+			+					+					+			
*Phormidium uncinatum*			+															
*Nostoc* sp.			+ *		+								+ *					
*Anabaena circinalis*					+													
*Anabaena oscillarioides*					+													
*Chroococcus*						+												
*Anabaechopsis circularis*						+												
*Spirulina laxissima SAG 256.80*										+								
*Planktothrix rubescens*								+ *					+ *					+
*Alkalinema pantanalense*																		+
*Gloeocapsa* sp.									+ *		+							
*Arthrospira* sp. str *PCC8005*			+ *															

Notes: + FlowCam analysis and + Molecular techniques with toxic genes * expression.

**Table 2:** Results from the BLAST search showing the similarity between the GenBank sequences and the sample sequences from this study. The families of each species are shown in a separate column.

Samples	Similarity %	Species Similar to	Family	Accession No
S2	93	Uncultured *Leptolyngbya* sp. Clone	Leptolyngbyaceae	KM108695.1
S3	94	*Synechocystis PCC 6803*	Oscillatoriophycideae	CP012832.1
S5	97	*Anabaena oscillarioides*	Nostocaceae	AJ630428.1
S7	99	*Synechocystis* sp. *PCC 6803*	Oscillatoriophycideae	CP012832.1
S8	99	*Leptolyngbya boryana*	Leptolyngbyaceae	AP014642.1
S9	97	*Synechocystis PCC 6803*	Oscillatoriophycideae	CP012832.1
S9	100	*Cylindrospermopsis raciborskii CHAB3438*	Oscillatoriophycideae	KJ139743.1
S9	100	*Aphanizomenon* sp.	Nostocaceae	GQ385961.1
S9	100	*Raphidiopsis curvata*	Nostocaceae	KJ139745.1
S10	96	*Spirulina laxissima SAG 256.80*	Spirulinaceae	DQ393278.1
S11	87	Uncultured Cyanobacterium clone	-	AM159315.1
S12	83	Uncultured Cyanobacterium clone	-	HQ189039.1
S13	90	Uncultured Cyanobacterium clone	-	JX041703.1
S14	98	*Leptolyngbya boryana*	Leptolyngbyaceae	AP014642.1
S16	83	*Leptolyngbya*	Leptolyngbyaceae	KJ654311.1
S18	96	*Alkalinema pantanalense*	Pseudanabaenaceae	KF246497.2

**Table 3:** Results from the BLAST search showing the similarity between the GenBank sequences and sample sequenced using PKS and HEP primers for toxin gene identification.

Primers	Sample No	Similarity %	Species Similar to	Accession No
PKS	S3	100	*Aphanizomenon* sp. *10E6*	GQ385961.1
	S3	100	*Raphidiopsis curvata*	KJ139745.1
	S3	100	*Cylindrospermopsis raciborskii*	AF160254.1
	S3	100	*Arthrospira* sp. *str. PCC 8005*	FO818640.1
	S3	100	*Nostoc* sp. *NIES-4103*	AP018288.1
	S9	93	*Calothrix* sp. *336/3*	CP011382.1
	S9	89	*Oscillatoria nigro-viridis PCC 7112*	CP003614.1
	S9	100	*Gloeocapsa* sp. *PCC 7428,*	CP003646.1
	S9	100	*Cylindrospermum* sp. *NIES-4074*	AP018269.1
HEP	S8	100	Uncultured *Microcystis* sp. *clone msp microcystin synthetase E (mcyE) gene, partial cds*	KF687998
	S8	100	*Microcystis panniformis FACHB-1757*	CP011339.1
	S8	100	*Microcystis aeruginosa PCC 7806*	AF183408.1
	S8	100	*Nostoc* sp. *152*	KC699835.1
	S8	100	*Planktothrix rubescens NIVA-CYA 98*	AM990462.1
	S13	100	*Nostoc* sp. *152*	KC699835.1
	S13	100	*Planktothrix rubescens NIVA-CYA 98*	AM990462.1
	S13	100	Uncultured *Microcystis* sp. *from Uganda*	FJ429839.2
	S13	100	*Microcystis aeruginosa PCC 7806SL*	CP020771.1
	S13	100	Uncultured *Microcystis* sp. *clone mw microcystin synthetase E (mcyE) gene, partial cds*	KF687997.1

**Table 4:** Divergence matrix for reflection of similarity.

	S2	S3	S5	S7	S8	S9	S10	S11	S12	S13	S14	S16	S18
Notwane River (S2)	‒												
Sand River (S3)	0.216	‒											
Nzhelele River (S5)	0.191	0.187	‒										
Mokolo River (S7)	0.167	0.064	0.130	‒									
Crocodile River (S8)	0.166	0.160	0.149	0.119	‒								
Nzhelele River (S9)	0.184	0.095	0.152	0.028	0.140	‒							
Sand River (S10)	0.155	0.216	0.153	0.156	0.169	0.169	‒						
Crocodile River (S11)	0.257	0.295	0.280	0.244	0.278	0.254	0.236	‒					
Nzhelele River (S12)	0.391	0.394	0.365	0.351	0.350	0.361	0.364	0.492	‒				
Mzingwane River (S13)	0.190	0.180	0.184	0.130	0.134	0.139	0.168	0.267	0.377	‒			
Shashe River (S14)	0.173	0.163	0.156	0.119	0.006	0.140	0.173	0.278	0.355	0.134	‒		
Musina borehole (S16)	0.376	0.359	0.312	0.314	0.342	0.321	0.343	0.414	0.555	0.371	0.347	‒	
Musina borehole (S18)	0.183	0.184	0.179	0.136	0.128	0.150	0.186	0.285	0.366	0.173	0.131	0.348	‒

**Table 5:** The location of sample sites and sample codes.

*River Names*	Samples Numbers
Limpopo River (Groblers’ bridge)	S1
Notwane River (Odi Bridge-Matabeleng)	S2
Sand River upstream	S3
Mogalakwena River next to Tolwe	S4
Mawoni River downstream Makhado oxidation ponds	S5
Lephalale river	S6
Mokolo River	S7
Crocodile River downstream Hartbeespoort dam	S8
Nzhelele River downstream near Tshipise	S9
Sand River downstream (at bridge on N1 road towards Musina)	S10
Crocodile River downstream (near bridge on road D1235) near Thabazimbi	S11
Nzhelele River upstream near Mphephu resort (downstream of Siloam oxidation ponds)	S12
Mzingwane River (Zimbabwe)	S13
Shashe River (near Irrigation scheme, Zimbabwe)	S14
Limpopo River next to Thuli coal mine	S15
Limpopo River abstraction point @ 0.0 m	S16
Limpopo River abstraction point @ 1.0 m	S17
Limpopo River abstraction point @ 1.68 m	S18

**Table 6:** The PCR primers used for amplification of 16S rRNA gene for cyanobacteria identification and for the amplification of genes related to cyanotoxins production. A—Individual annealing temperature, B—Reference annealing temperature, bp = base pairs.

Primers	Target Genes	Sequence (5′-3′)	A	B	Size (bp)	Amplified Gene	Ref.
27F 809R	-	AGAGTTTGATCCTGGCTCAG GCTTCGGCACGGCTCGGGTCGATA	52 64	60	780	16S rRNA	[85,86]
*mcyA-Cd* F *mcyA-Cd* R	*mcyA*	AAAATTAAAAGCCGTATCAAA AAAAGTGTTTTATTAGCGGCTCAT	51 43	59	297	Microcystin synthetase	[83]
HEPF HEPR	*mcyE/ndaF*	TTTGGGGTTAACTTTTTTGGGCATAGTC AATTCTTGAGGCTGTAAATCGGGTTT	57 55	52	472	Microcystin/nodularin synthetase	[81]
PKS M4 PKS M5	*cyr*	GAAGCTCTGGAATCCGGTAA AATCCTTACGGGATCCGGTGC	52 56	55	650	Cylindrospermopsin polypeptide synthase	[84]
M13 M14	*ps*	GGCAAATTGTGATAGCCACGAGC GATGGAACATCGCTCACTGGTG	57 57	55	597	Cylindrospermopsin peptide synthetase	[84]
